# The major thylakoid protein kinases STN7 and STN8 revisited: effects of altered STN8 levels and regulatory specificities of the STN kinases

**DOI:** 10.3389/fpls.2013.00417

**Published:** 2013-10-21

**Authors:** Tobias Wunder, Wenteng Xu, Qiuping Liu, Gerhard Wanner, Dario Leister, Mathias Pribil

**Affiliations:** ^1^Plant Molecular Biology (Botany), Department Biology I, Ludwig-Maximilians-Universität MünchenPlanegg-Martinsried, Germany; ^2^Ultrastrukturforschung, Department Biology I, Ludwig-Maximilians-Universität MünchenPlanegg-Martinsried, Germany; ^3^PhotoLab Trentino - a Joint Initiative of the University of Trento (Centre for Integrative Biology) and the Edmund Mach Foundation (Research and Innovation Centre)San Michele all'Adige (Trento), Italy; ^4^Mass Spectrometry Unit, Department Biology I, Ludwig-Maximilians-Universität MünchenPlanegg-Martinsried, Germany

**Keywords:** chloroplast, protein phosphorylation, PSII supercomplexes, redox, STN kinases, thylakoid ultrastructure

## Abstract

Thylakoid phosphorylation is predominantly mediated by the protein kinases STN7 and STN8. While STN7 primarily catalyzes LHCII phosphorylation, which enables LHCII to migrate from photosystem (PS) II to PSI, STN8 mainly phosphorylates PSII core proteins. The reversible phosphorylation of PSII core proteins is thought to regulate the PSII repair cycle and PSII supercomplex stability, and play a role in modulating the folding of thylakoid membranes. Earlier studies clearly demonstrated a considerable substrate overlap between the two STN kinases, raising the possibility of a balanced interdependence between them at either the protein or activity level. Here, we show that such an interdependence of the STN kinases on protein level does not seem to exist as neither knock-out nor overexpression of STN7 or STN8 affects accumulation of the other. STN7 and STN8 are both shown to be integral thylakoid proteins that form part of molecular supercomplexes, but exhibit different spatial distributions and are subject to different modes of regulation. Evidence is presented for the existence of a second redox-sensitive motif in STN7, which seems to be targeted by thioredoxin *f*. Effects of altered STN8 levels on PSII core phosphorylation, supercomplex formation, photosynthetic performance and thylakoid ultrastructure were analyzed in *Arabidopsis thaliana* using STN8-overexpressing plants (oe*STN8*). In general, oe*STN8* plants were less sensitive to intense light and exhibited changes in thylakoid ultrastructure, with grana stacks containing more layers and reduced amounts of PSII supercomplexes. Hence, we conclude that STN8 acts in an amount-dependent manner similar to what was shown for STN7 in previous studies. However, the modes of regulation of the STN kinases appear to differ significantly.

## Introduction

In *Arabidopsis thaliana*, the thylakoid kinase STN8 is predominantly responsible for the quantitative phosphorylation of PSII core proteins (CP43, D1, D2, and PsbH), particularly under high light conditions (Bonardi et al., 2005; Vainonen et al., 2005; Tikkanen et al., 2010). However, inactivation of STN8 alone does not completely abolish PSII core protein phosphorylation: D1 and D2 phosphorylation falls to about 50–60 and 35% of the wild-type level respectively (Vainonen et al., 2005). Only the knock-out of both STN8 and the LHCII kinase STN7 leads to quantitative loss of thylakoid phosphorylation, as monitored by immunodetection (Bonardi et al., 2005; Tikkanen et al., 2008). However, based on MS analyses, Fristedt et al. (2009) were still able to detect residual light-independent D2 phosphorylation in *stn7 stn8* double mutants, corresponding to less than 10% of the wild-type level. These results reveal a degree of overlap in substrate specificity between STN7 and STN8, although their main targets differ, and suggest that they might act in parallel rather than in series (Bonardi et al., 2005). By combining affinity chromatography with mass spectrometry, Reiland et al. (2011) have identified additional substrates of STN8, including the PGR5-like protein 1A (PGRL1A), which is essential for antimycin A (AA)-sensitive cyclic electron flow (CEF) around photosystem I (DalCorso et al., 2008). The differential phosphorylation of PGRL1A in *stn8-1* mutant plants is thought to permit more rapid switching between CEF and linear electron flow (LEF) during dark-light transitions (Reiland et al., 2011). Nevertheless, the function of reversible PSII core phosphorylation, the quantitatively major task of STN8, remains ambiguous.

Initially, the phosphorylated version of photo-damaged D1 was shown to be resistant to proteolysis (Koivuniemi et al., 1995), with the respective PSII complexes being able to move laterally from grana to stroma lamellae for subsequent dephosphorylation, degradation and replacement of damaged D1 (Rintamäki et al., 1996). The emerging model suggested that the intensity of PSII core protein phosphorylation was correlated with the increase in damage to PSII reaction centers (D1) as light intensity rises, which would be compatible with an involvement of STN8 in D1 turnover during photoinhibition (Baena-Gonzalez et al., 1999). Making use of the STN kinase mutant collection in *A. thaliana*, a later study performed by Bonardi et al. (2005) indicated that STN8-mediated phosphorylation of D1 *per se* is not essential for D1 turnover and PSII repair, challenging the concept that phosphorylation plays a major role in the degradation of D1. Further investigations again provided evidence that lack of STN8 is associated with greater susceptibility to photoinhibition (Nath et al., 2007) and revealed that D1 degradation is delayed in *stn8* and *stn7 stn8* mutants exposed to less intense high-light conditions (Tikkanen et al., 2008). Tikkanen et al. (2008) attribute this difference between WT and the *stn8* and *stn7 stn8* mutants to disturbances in the disassembly of PSII supercomplexes, leading to less efficient exchange of damaged D1 between grana and stroma lamellae due to changes in its migration behavior. More recent studies by Fristedt et al. (2009) confirmed the observed delay in D1 degradation in *stn8* and *stn7 stn8* plants and proposed that the observed increase in the diameter and density of stacked thylakoid membranes (grana) in these lines reduces lateral diffusion of proteins, including photo-damaged D1 and the bulky FtsH complex. The latter is responsible for D1 degradation (Nixon et al., 2005; Adam et al., 2006) and was reported to be spatially separated from PSII in STN8-deficient mutants due to its relocation from the dense grana to the stroma lamellae and grana margins (Fristedt et al., 2009). Therefore, phosphorylation of PSII core proteins is currently assumed to modulate the macroscopic rearrangement of the thylakoid membrane network, as well as the formation of PSII supercomplexes, and to affect lateral movement of proteins within the membrane, thus exerting its effects on D1 turnover indirectly.

In the present study, the effect of increased PSII core phosphorylation in a line overexpressing STN8 (oe*STN8*) was analyzed with respect to its impact on PSII supercomplex formation and modulation of thylakoid membrane structure. Furthermore, we analyzed the topology and localization of the STN kinases and show that neither knock-out nor overexpression of STN7 or STN8 affects the accumulation of the other. Finally, we show that STN8 protein levels in wild-type plants do not depend on ambient light conditions, and present evidence for a direct interaction of STN7 with thioredoxins, which is independent of its N-terminal cysteine motif.

## Materials and methods

### Plant material

The *Arabidopsis thaliana* L. (*A. thaliana*) ecotype Columbia-0, used in this study as wild type (WT), was obtained from NASC (Nottingham Arabidopsis Stock Center; accession number N1092). Previously described mutant lines employed in this study were *stn7-1, stn8-1, stn7 stn8* (Bonardi et al., 2005), oe*STN7*, STN7_C→S:65 + 70_ (Wunder et al., 2013), *hcf136* (Meurer et al., 1998), *psad1-1 psad2-1* (Ihnatowicz et al., 2004), *atpd-1*, and *petc-1* (Maiwald et al., 2003).

### Generation of STN8 overexpressor lines (oeSTN8)

To generate oe*STN8* lines, the full-length CDS of *STN8* was cloned into the plant vector pLeela, which is a derivative of pJawohl3-RNAi (GenBank Accession No. AF404854) containing a GATEWAY cassette introduced into the HpaI site, using the primers Stn8_attB1_ACC_f (GGGGACAAGTTTGTACAAAAAAGCAGGCTCTACCATGGCCTCTCTTCTCTCTC) and Stn8_attB2_Stop_r (GGGGACCACTTTGTACAAGAAAGCTGGGTTTCACTTGCTGAAACTGAGCTT). The *STN8*-pLeela construct containing a double Cauliflower Mosaic Virus (CMV) 35S promoter was introduced into the *stn8-1* mutant background via the floral-dip method (Clough and Bent, 1998). Plants were selected based on their BASTA resistance, segregation analysis was performed, and independent lines carrying a single T-DNA insertion locus were identified. Lines overexpressing the STN8 kinase (oe*STN8*) were identified by Western blot analysis employing an STN8-specific antibody.

### Growth conditions and light treatments

If not stated otherwise, plants to be analyzed were grown for 6 weeks under controlled conditions in a growth chamber on an 8 h/16 h day/night regime providing 100 μmol photons m^−2^s^−1^ during the light phase (standard lighting conditions). For experiments with the mutants *hcf136*, *petc-1, psad1-1 psad2-1*, and *atpd-1*, plants were grown on 1× MS medium including vitamins (Duchefa^®^) at 50 μmol photons m^−2^s^−1^. To study the effects of altered light conditions, plants were adapted to different light conditions specified as follows: 18h dark adaptation (D), adaptation to low light at 60–80 μmol photons m^−2^s^−1^ (LL) or high light at 800–1,200 μmol photons m^−2^s^−1^ (HL). The light source used for HL conditions was an Osram Powerstar HQIBT-D/400W lamp. Far-red light (FR) was emitted by LEDs at a wavelength of 740 nm and an intensity of 3.0 μmol photons m^−2^s^−1^. Adaptation to PSI and PSII light was performed essentially as described previously (Wagner et al., 2008). Briefly, 3-week-old plants were transferred from a climate chamber to either PSI- or PSII-specific light conditions. PSI light (15 μmol m^−2^s^−1^) was generated by clamping a medium red foil (Lee Filters, 027 Medium Red, transmittance 50% at 650 nm) over red fluorescent lamps (39 W) from Osram. PSII light (15 μmol m^−2^s^−1^) was generated by wrapping an orange foil (Lee Filters, 405 Orange, transmittance 50% at 560 nm) over white fluorescent lamps of the same type.

### Isolation of total proteins

Total protein extracts were prepared from 6-week-old leaves according to Haldrup et al. (1999). About 0.1 g of leaf material was homogenized in 200 μl solubilization buffer (100 mM Tris pH 8.0, 50 mM EDTA pH 8.0, 0.25 M NaCl, 1 mM DTT, 0.7% SDS) and heated to 65°C for 10 min. Samples were centrifuged for 10 min at 10,000 *g* to remove insoluble debris and the protein concentration in the supernatant was determined with the amido black assay described by Schaffner and Weissmann (1973). The ubiquitously expressed actin protein was used as a loading control.

### Isolation of thylakoid membranes

Thylakoids were isolated by a modified procedure based on Bassi et al. (1995). Briefly, leaf material from *A. thaliana* plants was homogenized in ice-cold isolation buffer (0.4 M sorbitol, 0.1 M Tricine-KOH pH 7.8, 0.5% milk powder, 20 mM NaF), filtered through two layers of Miracloth (Calbiochem) and centrifuged at 1,500 *g* for 10 min at 4°C. The membrane pellet was resuspended in ice-cold resuspension buffer (20 mM HEPES-KOH pH 7.5, 10 mM EDTA, 20 mM NaF) followed by a centrifugation step at 10,000 *g* for 10 min at 4°C after 10 min of incubation on ice. Thylakoids were resuspended in TMK buffer (10 mM Tris-HCl pH 6.8, 10 mM MgCl_2_, 20 mM KCl, 20 mM NaF). The chlorophyll concentration was determined in aqueous 80% acetone according to Porra (2002).

### Immunoblot analysis

If not stated otherwise, antibodies raised against specific epitopes of STN7 and STN8 were used for Western blot analysis in this study. The peptides CKKVKVGVRGAEEFG of STN8 and LQELREKEPRKKANAQ, located at the C-terminus of STN7, served as immunogens (BioGenes GmbH, Berlin, Germany). Immunoblot analyses with these antibodies, as well as phosphothreonine-specific antibodies (Cell Signaling Technology, Inc., Boston, USA) and polyclonal antibodies raised against actin (Dianova, Germany), RbcL, PsaC, PsaB, PsbO, PsaE, AtpB, Lhcb2, Lhca3 (all Agrisera, Sweden), were performed as previously described (Ihnatowicz et al., 2008).

### PAGE analyses

For Blue-native polyacrylamide gel electrophoresis (BN-PAGE), samples of freshly isolated thylakoids corresponding to 50 μg Chl were resuspended in solubilization buffer (750 mM ε-aminocaproic acid, 50 mM Bis-Tris pH 7.0, 5 mM EDTA pH 7.0, 50 mM NaCl) and solubilized for 60 min with 1.5% (w/v) digitonin or for 10 min with n-dodecyl-β-D-maltoside (β-DM) (Sigma) on ice (Pribil et al., 2010). Soluble was then separated from insoluble material by centrifugation (13,100 *g*, 4°C) for either 70 min (digitonin) or 10 min (β-DM). After supplementing with 5% Coomassie brilliant blue G-250 in 750 mM ε-aminocaproic acid, the solubilized material was fractionated by non-denaturing BN-PAGE (4–12% PA) at 4°C as outlined in Heinemeyer et al. (2004). For the second dimension separation, a single lane of the BN gel was incubated in 2× Laemmli buffer with 100 mM DTT for 30 min, then placed on top of a SDS gel and subjected to electrophoresis (two-dimensional (2D) BN/SDS-PAGE) (Schottkowski et al., 2009a,b). Standard 12% SDS-PAGE was performed according to Laemmli (1970) unless indicated otherwise. For non-reducing SDS-PAGE, reducing agents (like DTT) were omitted from the loading dye and samples were not boiled if not otherwise stated.

### Thylakoid fractionation after state 1 and 2 adaptation

Plants were acclimated to either state 1 or state 2 light and thylakoid fractionation was performed as previously described (Shapiguzov et al., 2010). Briefly, isolated thylakoids at a concentration of 0.6 mg of chlorophyll/mL were solubilized with 1% digitonin for 5 min, followed by stepwise centrifugation of supernatants. Pellets collected after centrifugation at 10,000 *g*, 40,000 *g* and 150,000 *g* represent enriched grana, grana margins and stroma lamellae fractions, respectively. The samples were analyzed by SDS-PAGE and Western blotting.

### Sucrose-gradient fractionation of thylakoid protein complexes

To prepare sucrose gradients, 11-mL aliquots of 0.4 M sucrose, 20 mM Tricine-NaOH (pH 7.5), 0.06% β-DM were subjected to three successive freeze-thaw (4°C) cycles. The gradient was underlaid with a cushion of 1 mL of 60% (w/v) sucrose. Thylakoids, prepared from plants that had been exposed to low light were washed twice with 5 mM EDTA (pH 7.8) and diluted to a final chlorophyll concentration of 2 mg/mL. Solubilization with β-DM at a final concentration of 1% was performed on ice for 10 min and followed by centrifugation (16,000 *g*, 5 min, 4°C). The supernatant was loaded on sucrose gradients and centrifuged at 132,000 *g* for 21 h at 4°C in a swing-out rotor (Beckman SW 40). Gradients were divided into 16 fractions, which were electrophoresed on a 15% SDS-PA gel and analyzed on a Western blot.

### Chloroplast isolation and fractionation into stroma and thylakoids

Chloroplasts were isolated from *A. thaliana* leaves as described (Aronsson and Jarvis, 2002). To obtain thylakoid and stroma fractions, chloroplasts were ruptured by adding 10 volumes of lysis buffer (20 mM HEPES-KOH pH 7.5, 10 mM EDTA) and incubating on ice for 30 min. The supernatant and pellet collected after a 30-min centifugation at 42,000 *g* and 4°C were used as stroma and thylakoid fractions, respectively.

### Salt washes of thylakoid membranes

Salt washes of thylakoid membranes were basically performed according to Karnauchov et al. (1997). Freshly isolated thylakoids (chlorophyll concentration 0.5 mg/mL) were incubated for 30 min on ice in HS buffer (0.1 M sucrose, 10 mM HEPES-NaOH pH 8.0) or HS buffer containing 2 mM NaCl, 2 M NaBr, 2 M NaSCN, 0.1 M Na_2_CO_3_ or 0.1 M NaOH, respectively. After addition of two volumes of HS buffer, the samples were centrifuged at 13,100 *g* for 15 min at 4°C. Proteins in the pellet fraction were subsequently solubilized directly in Laemmli buffer, whereas the supernatant was first precipitated in 80% acetone.

### Chlorophyll fluorescence measurements during light induction and PSII inactivation induced by high light

Steady-state photosynthetic parameters were measured under actinic red light of increasing light intensity (22, 37, 53, 95, 216, 513, 825, 1,287, and 1,952 μmol photons m^−2^s^−1^), using the Dual-PAM 100 system (Heinz Walz GmbH, Effeltrich, Germany) in the Dual PAM mode, according to the manufacturer's instructions and with standard settings. Plants were dark-adapted for 10 min prior to measurements and allowed to adapt for 5 min to each new light level. Five plants of each genotype were analyzed for each measurement using always the sixth true leaf of the respective plant. Photoinhibition of photosystem II (PSII) was induced over a period of 10 h with aid of the Imaging PAM System (Walz) by exposing leaves to blue light alternating every 2 min between HL (1,250 μmol photons m^−2^s^−1^) and LL (10 μmol photons m^−2^s^−1^). Maximum PSII quantum yield [Fv/Fm = (Fm − Fo)/Fm] was determined every 60 min after the LL phase and an additional 5-min dark adaptation.

### Redox titration of STN7 in thylakoid membranes

Thylakoid proteins were isolated and equilibrated on ice for 3 h with various redox buffers (100 mM MOPS pH 7.0, 330 mM sorbitol) containing DTTred and DTTox in various molar ratios. Reactions were solubilized with 2% SDS and subsequently separated by non-reducing 15% SDS-PAGE. After transfer of proteins to PVDF membrane, reduced and oxidized forms of STN7 were detected by immunoblot analysis.

### Thioredoxin (TRX) affinity purification

Affinity purification was basically performed as described by Motohashi et al. (2001). His-tagged recΔ TRX-f (-m) was expressed in *E. coli* and purified by Ni-NTA resin according to the Qiagen protocol for native protein purification (Expressionist, Qiagen), without eluting resin-bound proteins. An aliquot of isolated thylakoid membranes (=1 mg Chl) was solubilized with 1.5% digitonin in 50 mM Tris-HCl pH 8.0 for 60 min. After centrifugation at 16,100 *g* for 70 min, the supernatant was incubated with the TRX-coupled resin (~5 mg TRX/mL) for 60 min at RT. The column was washed three times with washing buffer (50 mM Tris-HCl pH 8.0, 200 mM NaCl, 0.2% digitonin) and proteins retained by thioredoxin were eluted by adding 10 mM DTT. Samples were analyzed on a Western blot, using STN7-specific antibodies.

### Mobility shift assay of TRX

For the TRX mobility shift assay, 10 μg of thylakoids were solubilized with 0.2% deoxycholic acid (DOC) and incubated with 25 μg of recombinant TRX-f (recΔ TRX-f) for 30 min in 100 mM MOPS (pH 7.0) and 330 mM sorbitol at RT. Untreated thylakoids (in 0.2% DOC buffer) served as a control. Subsequently, protein mixtures were subjected to non-reducing SDS-PAGE and immunoblotting.

### Analysis of thylakoid membrane ultrastructure by transmission electron microscopy (TEM)

Plants were grown for 4 weeks in the climate chamber on a 12 h/12 h day/night regime. 1.5 h after the onset of the light phase the sixth rosette leaf was cut off and fixed for 1 h with 2.5% glutaraldehyde in fixation buffer (75 mM cacodylic acid, 2 mM MgCl_2_ pH 7.0). The material was washed with buffer, incubated for 2 h with 1% osmium tetroxide in fixation buffer, washed again with fixation buffer and finally with distilled water. Samples were dehydrated by sequential incubation in increasing acetone concentrations, embedded in resin and sectioned with a microtome after complete polymerisation. Micrographs of the sections were taken with an EM-912 electron microscope (Zeiss) equipped with an integrated OMEGA energy filter operated in the zero-loss mode.

## Results

### STN7 and STN8 are not linked by a feedback loop

**Figure 1:**
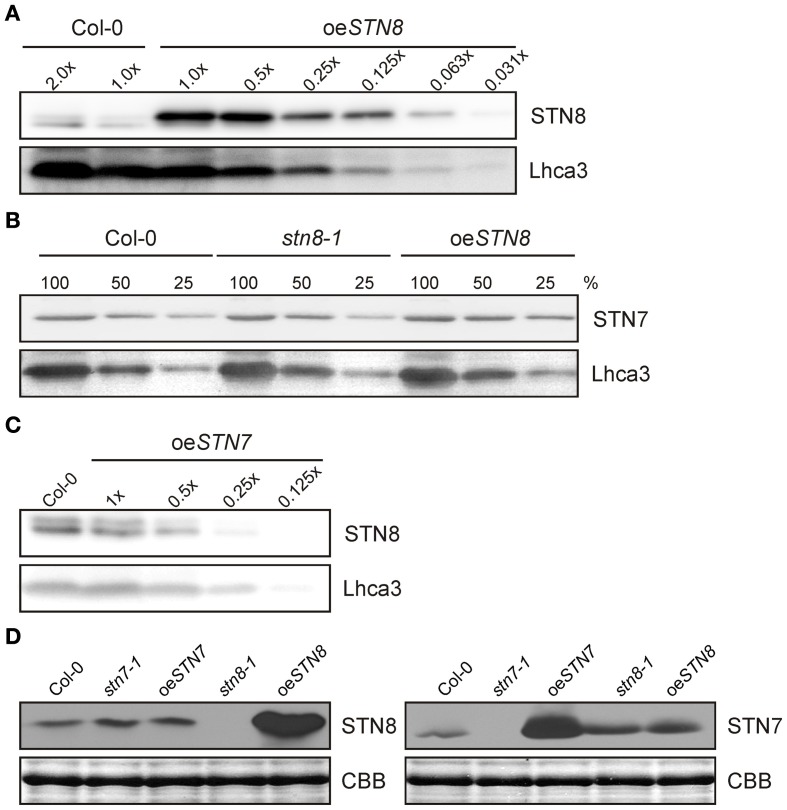
**Non-interdependence of STN kinase accumulation. (A)** WT (Col-0) thylakoid proteins corresponding to 10 μg (2.0×) and 5 μg (1.0×) of Chl and serial dilutions of oe*STN8* thylakoids were loaded to quantify the levels of STN8 protein in oe*STN8* plants. Proteins were subjected to SDS-PAGE and Western blot analysis using STN8- and Lhca3-specific antibodies. **(B)** STN7 accumulation in thylakoids of WT (Col-0), oe*STN8* and *stn8-1* mutant plants. Thylakoid proteins, corresponding to 8 μg (100%), 4 μg (50%), and 2 μg (25%) of Chl, from each genotype were loaded on a SDS gel and probed using specific antibodies against STN7 and Lhca3 (as loading control). **(C)** Accumulation of STN8 in thylakoids of WT (Col-0) and oe*STN7*. Thylakoids of WT corresponding to 5 μg of Chl and serial dilutions of oe*STN7* thylakoid proteins were separated by SDS-PAGE, and immunoblots were probed with antibodies specific for STN8 and Lhca3 (as loading control). **(D)** Thylakoids of WT (Col-0), *stn8-1*, *stn7-1*, oe*STN8*, and oe*STN7* plants corresponding to 5 μg of Chl were analyzed by Western blotting with antibodies raised against an STN8-specific (left panel) or an STN7-specific (right panel) peptide fragment. Replicate SDS-gels were stained with Coomassie brilliant blue (CBB) and the section with the LHCII signal is shown as loading control.

Previous studies had demonstrated a significant substrate overlap between the kinases STN7 and STN8 (Bonardi et al., 2005). To investigate whether alterations in STN7 levels affect STN8 accumulation and *vice versa*, *A. thaliana* mutant plants either lacking (*stn7-1* and *stn8-1*) or overexpressing (oe*STN7* and oe*STN8*) one of the kinases were analyzed. To this end, STN8 overexpressor lines (oe*STN8*) were generated that express STN8 under the control of the 35S promoter in the *stn8-1* mutant background. This resulted in an increase of 16-fold or more in STN8 protein levels relative to the WT (Figure 1A). The analysis of STN7 and STN8 proteins in oe*STN8* plants and the previously described mutant lines *stn7-1*, *stn8-1* and oe*STN7* (Bonardi et al., 2005; Wunder et al., 2013) using epitope-specific STN antibodies (Figures 1B–D) revealed no significant alterations in the level of the genetically unperturbed kinase. Thus, fluctuations in the availability of one STN kinase do not lead to compensatory changes in the concentration of the other one.

### The STN kinases are thylakoid integral membrane proteins with distinct spatial distributions that form part of large protein complexes

**Figure 2:**
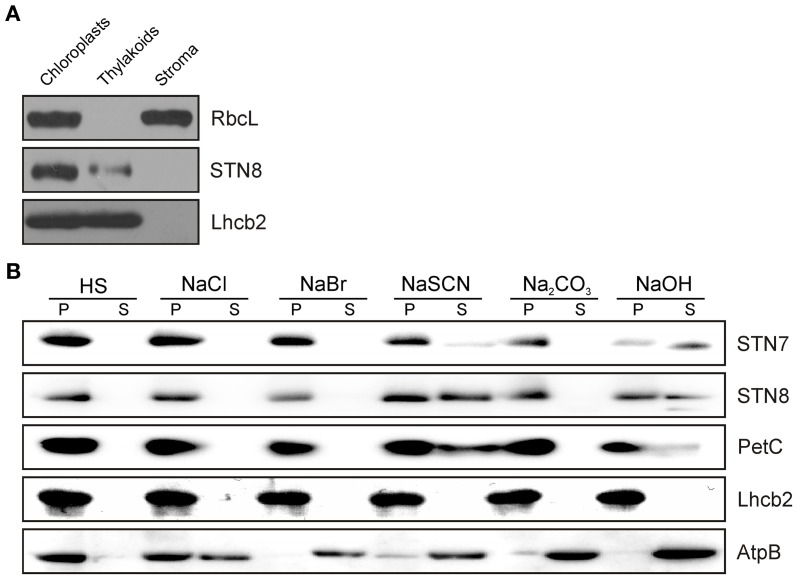
**Sub-organellar localization of STN8 and membrane association of the STN kinases. (A)** *Arabidopsis* WT chloroplasts were fractionated into soluble and membrane components and subsequently separated by SDS-PAGE. After Western transfer, STN8, RbcL (stroma marker) and Lhcb2 (thylakoid marker) were detected using specific antibodies. **(B)** Extraction of membrane-associated proteins with alkaline buffers or chaotropic salt solutions. Thylakoid membranes from WT plants were resuspended at 0.5 mg chlorophyll/mL in HS buffer containing either 2 M NaCl, 0.1 M NaBr, 2 M NaSCN, 0.1 M Na_2_CO_3_, 0.1 M NaOH, or no additive. After incubation on ice, samples were fractionated into soluble (S) and membrane-associated proteins (P) and immunolabeled with antibodies specific for STN7, STN8, AtpB (a representative peripheral membrane protein), PetC (a membrane protein with a single hydrophobic domain predominantly anchored by electrostatic interactions), or Lhcb2 (an integral membrane protein with three transmembrane helices).

Previous evidence for the localization of the STN8 kinase was based on *in-vitro* import studies with pea chloroplasts (Bonardi et al., 2005). However, localization studies of STN8 with specific antibodies have not been performed. For this purpose, WT *A. thaliana* chloroplasts were fractionated into soluble and membrane components and subjected to immunoblot analysis using antibodies raised against a defined STN8 epitope. STN8 was detected exclusively in the membrane fraction, ruling out the existence of a soluble variant of the protein (Figure 2A). The purity of the respective fractions was confirmed by the detection of stroma (RbcL) and thylakoid membrane (Lhcb2) marker proteins. To clarify whether the hydrophobic moieties of the STN kinases truly represent predicted transmembrane domains (TMDs) (Vainonen et al., 2005) or mediate extrinsic attachment to the thylakoid membrane only, WT thylakoids were treated with alkaline buffers or chaotropic agents. Both STN7 and STN8 behaved like the Rieske protein (PetC) (Figure 2B), which has been shown to possess a single hydrophobic domain and to associate with the thylakoid membrane predominantly via electrostatic interactions (Karnauchov et al., 1997). Therefore, we conclude that both STN kinases constitute integral membrane proteins as previously suggested (Vainonen et al., 2005; Lemeille et al., 2009).

**Figure 3:**
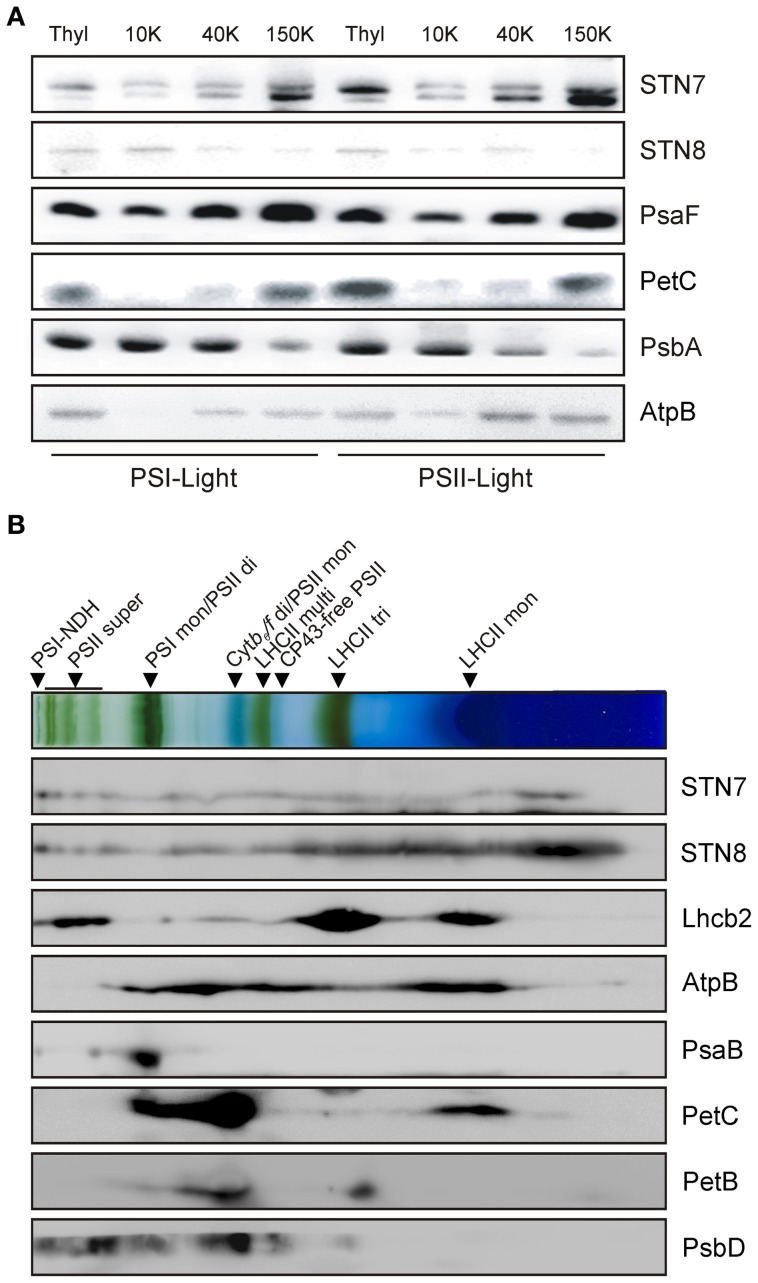
**Localization of the STN kinases within the thylakoid membrane. (A)** Analysis of STN7 and STN8 localization by thylakoid membrane sub-fractionation. Thylakoids of PSII- or PSI-light adapted WT plants were fractionated into grana lamellae (10 K), grana margins (40 K) and stroma lamellae (150 K) by differential ultracentrifugation after digitonin solubilization. Proteins were separated by SDS-PAGE and analyzed by immunolabeling using antibodies against STN7, STN8, PsaF, PetC, PsbA, and AtpB. **(B)** Analysis of STN7 and STN8 distribution by 2D BN/SDS-PAGE. Thylakoid membranes of WT equivalent to 50 μg of Chl were solubilized with β-DM, subjected to 2D BN/SDS-PAGE and analyzed by Western blotting. STN7, STN8 and various subunits of the major photosynthetic complexes (AtpB, Lhcb2, PsaB, PetC, PetB, and PsbD) were detected with specific antibodies. Protein-Chl complexes are indicated by black arrowheads. The bands detected were identified as specific protein complexes in accordance with previously published profiles (Armbruster et al., 2013): PSI-NDH supercomplex (PSI-NDH), PSII supercomplexes (PSII super), PSI monomers and PSII dimers (PSI mon and PSII di), PSII monomers and dimeric Cyt *b*_*6*_*/f* (PSII mon and Cyt *b*_*6*_*/f* di), PSII monomers w/o CP43 (CP43-free PSII), multimeric (LHCII multi), trimeric (LHCII tri), and monomeric (LHCII mon) LHCII.

To monitor possible changes in the sub-thylakoid localization of the STN kinases under either STN7/8-activating or -inhibiting light conditions, thylakoids from WT plants were isolated after exposure to PSI- or PSII-specific light, and fractionated via differential centrifugation after solubilization with 1.5% digitonin. While STN8 was enriched in the grana membranes (10K fraction), STN7 was predominantly located in the stroma lamellae (150K fraction) (Figure 3A). The distributions of STN7 and STN8 between the different membrane fractions were not affected by exposure to PSI- or PSII-favoring light conditions. These data indicate that the major fraction of STN8 resides within the grana stacks close to its assumed primary substrates - the subunits of PSII - irrespective of light conditions. The major proportion of STN7 was localized to the stroma lamellae, with only a minor fraction being found in the grana margins or grana stacks. The distribution of STN7 thus coincides with that of PetC, a subunit of the Cyt *b*_*6*_*f* complex known to interact physically with Stt7, the *Chlamydomonas* homolog of STN7 (Lemeille et al., 2009) (Figure 3A).

To further refine the localization of the STN kinases within the thylakoid membrane, their association with known thylakoid protein complexes was investigated. To this end, WT thylakoids were solubilized with 1.6% β-DM, separated on BN-PA gels and subsequently resolved via 2D-PAGE. Both STN7- and STN8-specific antibodies gave rise to signals covering the entire molecular weight range, from large supercomplexes down to the free protein fraction (Figure 3B), suggesting that they associate at least weakly with some of the major photosynthetic complexes.

Thylakoids were also fractionated by ultracentrifugation on a linear sucrose gradient after β-DM solubilization. Again, STN7 and STN8 were both identified in fractions containing high-molecular-weight (HMW) complexes (Figure S1A). These results agree with previous findings showing that Stt7 associates with a HMW complex (Lemeille et al., 2009), indicating that neither of the STN kinases normally occurs as a monomeric polypeptide, but remains in close association with HMW complexes.

The association of STN8 with HMW complexes was also probed by analyzing different mutant plants devoid of one of the major photosynthetic complexes, following analogous studies previously performed for STN7 (Wunder et al., 2013). Here, absence of PSII (in *hcf136*) also prevented STN8 accumulation, which is in agreement with their co-localization to grana lamellae (Figure S1B). Moreover, a strong decrease in STN8 levels was observed in plants lacking PSI (*psad1-1 psad2-1*), which is probably due to a significant concomitant reduction in PSII amounts rather than to the loss of PSI *per se*. Thus, the presence of PSII, the major constituent of thylakoid protein supercomplexes, seems to be a prerequisite for STN8 accumulation.

### Redox sensitivity of STN7

**Figure 4:**
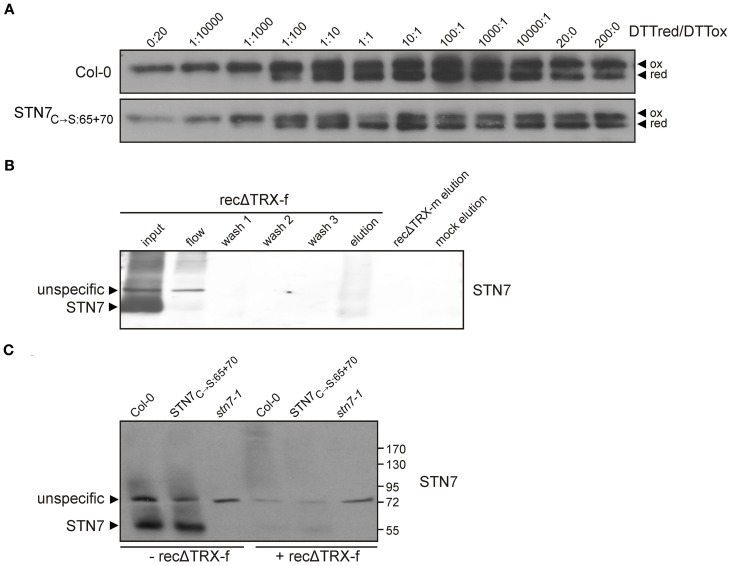
**Redox sensitivity of STN7. (A)** Redox titration of STN7 variants. Thylakoids of WT (Col-0) and STN7_C→S:65 + 70_ were equilibrated at various redox potentials using the DTTred/DTTox redox couple. After separation by non-reducing SDS-PAGE, STN7 was detected by immunoblotting. DTTred/DTTox ratios are indicated above each lane. Oxidized (ox) and reduced (red) forms of the STN7 monomer are indicated by black arrowheads. **(B)** STN7—TRX-f interaction assayed by thioredoxin affinity chromatography. His-tagged recombinant thioredoxins f and m, with one cysteine replaced by serine (recΔ TRX-f, -m), were bound to Ni-NTA and incubated with thylakoids solubilized with 1.5% digitonin corresponding to 2 mg of Chl. After several washes, the eluates from recΔ TRX-f and -m resins were subjected to Western blot analysis using STN7-specific antibodies. Solubilized WT (Col-0) thylakoids (input; corresponding to 10 μg of Chl), the flow through off the recΔ TRX-f resin (flow; equivalent to 10 μg Chl), washes 1–3 of the recΔ TRX-f resin and the eluate from a similarly treated Ni-NTA resin not coupled to recΔ TRX (mock elution) were loaded as controls. **(C)** TRX-f mobility shift assay. Thylakoid membranes (10 μg Chl) from WT (Col-0), STN7_C→S:65 + 70_ and *stn7-1* mutants were solubilized with 0.2% DOC and incubated with either 25 μg of recΔ TRX-f or without additives. Proteins were separated by non-reducing SDS-PAGE and Western analysis was performed using STN7-specific antibodies.

As recently demonstrated, STN7 activity is redox sensitive, and depends on an N-terminal cysteine motif (Wunder et al., 2013). To analyze possible redox-based effects on the tertiary structure of STN7, thylakoids of WT and STN7_C→S:65 + 70_ mutant plants devoid of the N-terminal redox-sensitive cysteine motif (Wunder et al., 2013) were incubated in a series of buffers containing reduced and oxidized DTT in different molar ratios, and then subjected to non-reducing SDS-PAGE. Interestingly, both wild-type STN7 and STN7_C→S:65 + 70_ monomers showed a clear size-shift in response to the redox treatment (Figure 4A), suggesting the occurrence of a redox-dependent conformational change in STN7 or the release of a possible redox-sensitive co-factor. This implies that the N-terminal CxxxxC motif is not the only redox-sensitive site in the STN7 protein. Under progressively more reducing conditions the amounts of STN7 detected increased significantly. This may indicate that under oxidizing conditions STN7 associates with various HMW aggregates/complexes, such that less STN7 enters the gel in the absence of reduced DTT. Also the STN7 epitope that is recognized by the peptide-specific antibody might be more accessible under reducing conditions.

STN7 activity, especially its inactivation under HL, is thought to be regulated via stromal thioredoxins such as TRX-f and -m (Rintamäki et al., 2000; Lemeille and Rochaix, 2010). So to search for a direct interaction between STN7 and TRX-f and/ or -m, recombinant TRX-f and TRX-m variants containing a single Cys-to-Ser exchange in their catalytic CGPC motifs (recΔ TRXs) were generated. Due to this single cysteine mutation in the conserved TRX motif, stable covalent bonds instead of a transient interaction are formed during the disulfide bridge interchange reaction with the substrate. In this way, potential targets of TRXs are trapped as stable dimeric intermediates. To confirm such covalent binding of STN7 to recΔ TRXs, WT thylakoids were solubilized with 1.5% (w/v) digitonin and incubated with either recΔ TRX-f or recΔ TRX-m immobilized on Ni-NTA resin. After several washing steps proteins interacting with recΔ TRX-f and -m were eluted with a DTT-containing buffer, and the presence of STN7 was determined via Western analysis. Although slightly shifted in size, STN7 could indeed be detected in the fraction eluted from the recΔ TRX-f resin, whereas no STN7 was detectable in the same fraction from the recΔ TRX-m column (Figure 4B). In addition, the notion that STN7 can covalently bind to recΔ TRX-f was supported by a significant decrease in amounts of STN7 in the flow-through in comparison to the input fraction (Figure 4B).

To further assess the possible interaction of STN7 with TRX-f, a TRX mobility-shift assay was performed. To this end, recΔ TRX-f was incubated with solubilized thylakoids from WT, *stn7-1* and STN7_C→S:65 + 70_ plants (Figure 4C). Covalent binding of recΔ TRX-f to STN7 is expected to reduce the electrophoretic mobility of the STN7 signal due to the increased molecular weight of the resulting complex, but no STN7-TRX-f linkage product was detectable by Western analysis. However, the STN7 monomer disappeared almost completely upon addition of TRX-f (Figure 4C), suggesting a direct interaction between the two. Whether TRX-f treatment led to precipitation of STN7 or to formation of cross-linked, HMW aggregates in which the STN7 epitope is inaccessible to the antibody could not be definitively clarified. Interestingly, the STN7_C→S:65 + 70_ variant behaved like wild-type STN7 (Figure 4C), which supports the inference from the redox titration assay (Figure 4A) regarding the existence of a second redox-sensitive site within STN7.

### STN8 protein levels do not respond to changes in light conditions

**Figure 5:**
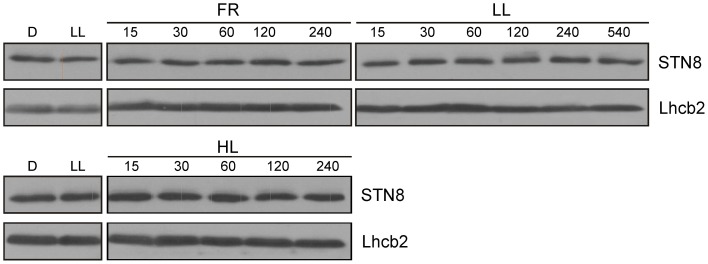
**STN8 protein accumulation upon exposure to D, LL, FR, and HL.** WT plants were dark-adapted for 18 h, transferred to low light (LL; 80 μmol photons m^−2^s^−1^) for 2 h and then to FR or HL (HL; 800 μmol photons m^−2^s^−1^) for an additional 4 h. Leaf material was collected after each treatment and thylakoids were fractionated by SDS-PAGE and analyzed using antibodies specific for STN8 and Lhcb2 (loading control).

STN8 activity was previously shown to be regulated by changes in light quality and quantity (Bonardi et al., 2005; Tikkanen et al., 2010). In particular, FR and HL treatments led to a decrease and increase in PSII core protein phosphorylation respectively (Tikkanen et al., 2008, 2010). To investigate whether these changes are due to alterations in STN8 level or activity, WT plants were exposed for 2 h to LL after 18 h of darkness and subsequently transferred for 4 h to FR or HL (800 μmol photons m^−2^s^−1^), after which the amounts of STN8 present were determined. In contrast to STN7 (Wunder et al., 2013), STN8 levels did not change markedly upon these light-shift treatments, and showed no significant variations even after exposure to LL for up to 9 h (Figure 5). This stable expression profile suggests that STN8 is regulated at the level of specific activity rather than amount.

### Overexpression of STN8 increases PSII protein phosphorylation and disassembly of PSII supercomplexes under HL conditions

**Figure 6:**
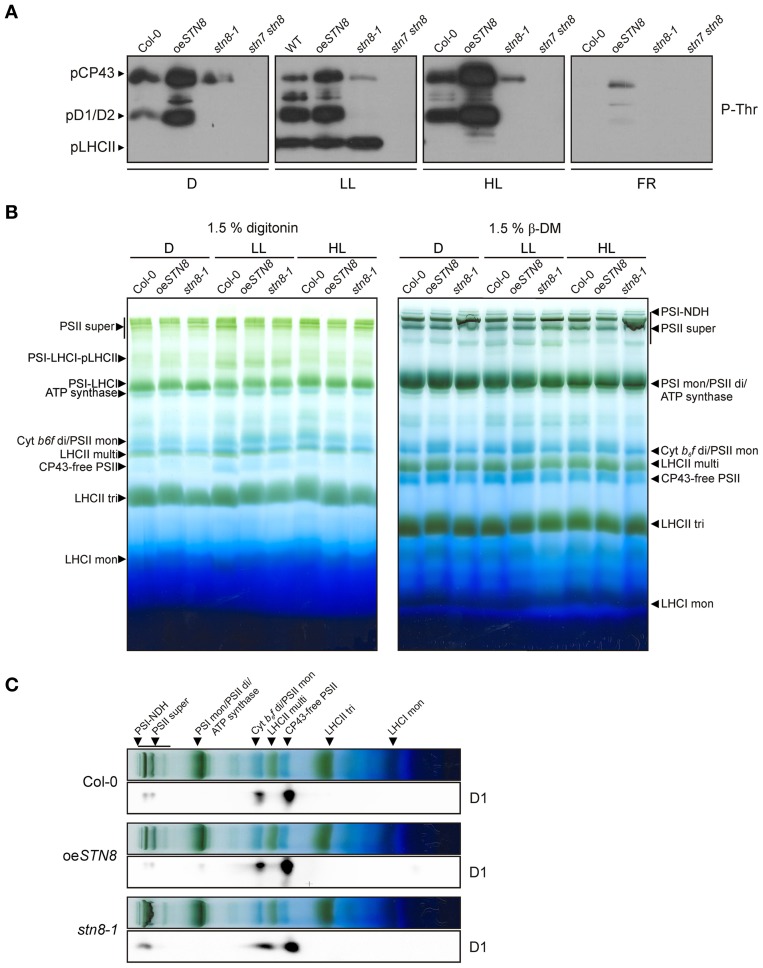
**Effects of altered STN8 levels on thylakoid phosphorylation and PSII supercomplex formation. (A)** Thylakoid protein phosphorylation in WT (Col-0), oe*STN8, stn8-1*, and *stn7 stn8* plants dark-adapted for 18 h (D), subsequently transferred to LL (80 μmol photons m^−2^s^−1^) for 2 h and for additional 2 h to FR or 4 h to HL (1,000 μmol photons m^−2^s^−1^). After these light treatments, isolated thylakoids were analyzed by immunodetected using phosphothreonine (pThr) -specific antibodies (Cell Signaling). The positions of phosphorylated LHCII (pLHCII), CP43 (pCP43) and D1/2 (pD1/2) are indicated by black arrowheads. **(B)** Patterns of protein complexes solubilized from WT (Col-0), oe*STN8* and *stn8-1* thylakoids and separated on BN-PA gels. Thylakoid membranes of WT, oe*STN8* and *stn8-1* plants exposed to either LL (80 μmol photons m^−2^s^−1^) or HL (1,200 μmol photons m^−2^s^−1^) for 2 h, or kept in the dark for 18 h, were solubilized with 1.5% digitonin (left panel) or 1.5% β-DM (right panel) and separated by BN-PAGE. Protein-Chl complexes are indicated by black arrowheads. The bands detected were identified as specific protein complexes in accordance with previously published profiles (Armbruster et al., 2013): PSI-LHCI-pLHCII supercomplex (PSI-LHCI-pLHCII), PSI-LHCI supercomplex (PSI-LHCI), PSI-NDH supercomplex (PSI-NDH), PSII supercomplexes (PSII super), PSI monomers and PSII dimers (PSI mon and PSII di), PSII monomers and dimeric Cyt *b*_*6*_*f* (PSII mon and Cyt *b*_*6*_*f* di), PSII monomers w/o CP43 (CP43-free PSII), multimeric (LHCII multi), trimeric (LHCII tri) and monomeric (LHCII mon) LHCII. **(C)** BN-PAGE lanes bearing HL-treated samples corresponding to those shown in the right panel of **(B)** were subjected to SDS-PAGE in the second dimension and probed with D1-specific antibodies.

To determine the effects of increased STN8 accumulation on thylakoid protein phosphorylation, WT, *stn8-1*, *stn7 stn8* and oe*STN8* plants were dark-adapted for 18 h, then exposed for 2 h to LL (80 μmol photons m^−2^s^−1^) followed by either 4 h of HL (1,000 μmol photons m^−2^s^−1^) or 2 h of FR. The phosphorylation status of isolated thylakoids was then monitored by Western blot using phosphothreonine-specific antibodies (Figure 6A). In general, oe*STN8* plants exhibited a significant increase in PSII core protein phosphorylation under all investigated light conditions. While PSII phosphorylation in WT plants decreased in the dark compared to LL conditions, PSII phosphorylation levels in oe*STN8* lines remained high under both conditions. After FR treatment, residual PSII phosphorylation was detectable only in oe*STN8*. The strongest increase in PSII core protein phosphorylation in oe*STN8* relative to WT was observed under HL, which is known to induce STN8 activity (Bonardi et al., 2005). Interestingly, pLHCII levels differed little between oe*STN8* and WT, suggesting that the excess STN8 present in oe*STN8* plants does not significantly affect the phosphorylation status of STN7-specific substrates under the light conditions examined (Figure 6A).

Under HL conditions, thylakoid phosphorylation - in particular phosphorylation of the PSII core proteins - has been shown to promote the disassembly of PSII supercomplexes (Tikkanen et al., 2008). To examine effects of increased PSII protein phosphorylation on PSII supercomplexes, BN-PAGE analyses were performed on thylakoids after exposure of oe*STN8* plants to 18 h D and 2 h LL (80 μmol photons m^−2^s^−1^) or 2 h HL (1,200 μmol photons m^−2^s^−1^). Solubilization of thylakoid samples from HL-treated plants with 1.5% digitonin prior to BN-PAGE revealed no significant differences between WT and *stn8-1*, while a slight decrease in the amount of PSII supercomplexes was noted in oe*STN8* (Figure 6B, left panel). Similar, but more pronounced, effects on PSII supercomplex accumulation were observed when thylakoids were solubilized with 1.5% β-DM. Here, WT plants exposed to HL once again contained more PSII supercomplexes than oe*STN8* and fewer than *stn8-1* (Figure 6B, right panel), which is in accordance with earlier reports (Tikkanen et al., 2008).

2D-PAGE analysis was performed on HL-treated samples to assess the distribution of the PSII core proteins between PSII supercomplexes, dimers and monomers. Greater accumulation of D1 in PSII supercomplexes was observed in *stn8-1* compared to WT, whereas the opposite was the case for oe*STN8* (Figure 6C). In oe*STN8* a clear shift in the ratio of PSII supercomplexes to PSII dimers and monomers in favor of the latter two was observed in comparison to the situation in WT and the *stn8-1* mutant. This suggests that disassembly of PSII supercomplexes is indeed enhanced by an increase in PSII core protein phosphorylation, as previously described (Tikkanen et al., 2008).

### Increased STN8 levels reduce susceptibility to PSII photoinhibition and lead to a moderate rise in the oxidation state of the PQ pool

**Figure 7:**
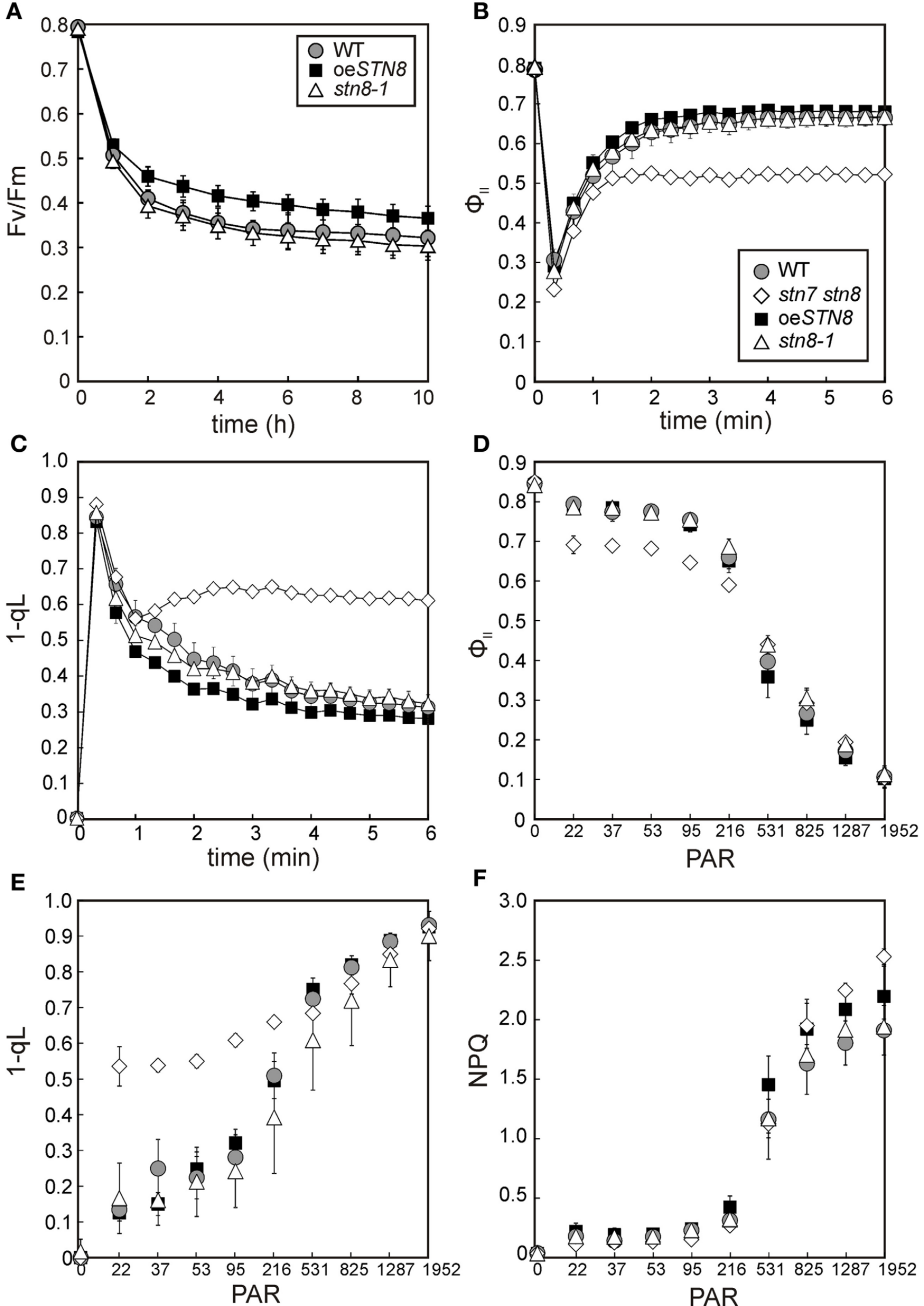
**Kinetics of Chl a fluorescence and time-course of PSII photoinhibition in WT, *stn8-1* and oe*STN8* plants. (A)** WT, *stn8-1* and oe*STN8* plants were exposed to fluctuating (blue) light intensities for 10 h by switching from HL (1,250 μmol photons m^−2^s^−1^) to LL (10 μmol photons m^−2^s^−1^) and back every 3 min. The maximum quantum yield of PSII (Fv/Fm) was measured every 60 min after the LL phase and additional 5 min dark adaptation. HL exposure and PAM measurements were performed using an Imaging PAM system (Heinz Walz GmbH). **(B,C)** Time-course of the effective quantum yield of PSII (Φ_II_) **(B)** and excitation pressure of PSII (1-qL) **(C)** of plants pre-incubated for 10 min in the dark and exposed to actinic red light (22 μmol photons m^−2^s^−1^) for 6 min. **(D–F)** Dependence of Chl a fluorescence on light intensity. The effective quantum yield of PSII (Φ_II_) **(D)**, the excitation pressure of PSII (1-qL) **(E)** and non-photochemical quenching of chlorophyll fluorescence (NPQ) **(F)** were monitored as red light intensity was increased stepwise at 5-min intervals (22, 37, 53, 95, 216, 513, 825, 1,287, and 1,952 μmol photons m^−2^s^−1^) after 10 min of dark adaptation. PAR, photosynthetically active radiation (μmol photons m^−2^s^−1^); circles with gray filling, WT; squares with black filling, oe*STN8*; triangles; *stn8-1*; diamonds, *stn7 stn8*. **(A–F)** Plants were grown under an 8 h/16 h day/night regime at 100 μmol photons m^−2^s^−1^ prior to measurements. Average values (±SD) of at least five individual plants are shown.

In previous studies, a decrease in the turnover of damaged D1 was observed in leaves of *stn7 stn8* and *stn8-1* plants exposed to high light. This was attributed to the lack of PSII core protein phosphorylation, which is a prerequisite for the disassembly of PSII supercomplexes and the migration of damaged PSII complexes from grana to stroma lamellae (Tikkanen et al., 2008; Fristedt et al., 2009). Accordingly, a reduction in PSII phosphorylation is expected to increase sensitivity to photoinhibition during HL treatment, which translates into lower Fv/Fm values relative to WT. To address the question whether increased PSII phosphorylation levels affect susceptibility to photoinhibition, WT, *stn8-1* and oe*STN8* plants were exposed to fluctuating light intensities by switching from LL (10 μmol photons m^−2^s^−1^) to HL (1,250 μmol photons m^−2^s^−1^) and back every 3 min. While *stn8-1* and WT plants did not differ significantly in their Fv/Fm values, as previously shown (Bonardi et al., 2005), Fv/Fm values in oe*STN8* plants were slightly higher than in WT (Figure 7A). This can be explained if one assumes that light fluctuations enhance the positive effect of elevated STN8 levels at the onset of HL by allowing a more rapid response to sudden light stress. Overall, enhanced disassembly of PSII supercomplexes under HL conditions, as in the case of oe*STN8* (Figure 6), seems to have only a moderate effect on PSII repair.

To explore general effects of altered PSII core phosphorylation on photosynthesis, Chl a fluorescence and absorption parameters were recorded for WT, oe*STN8*, *stn8-1* and *stn7 stn8* plants during dark-light transitions (Figures 7B,C). When dark-adapted plants were exposed to LL (22 μmol photons m^−2^s^−1^) for 6 min, no significant differences in the effective quantum yield of PSII (Φ_II_) were detected between WT and *stn8-1*, while in oe*STN8* plants the Φ_II_ was initially higher and gradually converged to WT levels during the course of the measurement (Figure 7B). 1-qL, a measure of the excitation pressure of PSII, was somewhat lower in oe*STN8* compared to WT and *stn8-1*, which suggests a slightly more oxidized PQ pool (Figure 7C). As expected, the *stn7 stn8* mutant showed higher 1-qL and lower Φ_II_ values than the WT. Due to the slightly increased resistance of oe*STN8* plants to photoinhibition (Figure 7A), a degree of alteration in photosynthetic performance could be expected even under short-term exposure to high light. However, upon stepwise increase in light intensity (in 5-min steps), Φ_II_ and 1-qL measurements revealed no significant differences between oe*STN8* and WT plants (Figures 7D–F). Non-photochemical quenching (NPQ) alone showed a slight increase in oe*STN8* under strong light intensities, as in the case of *stn7 stn8* (Figure 7F).

To assess the performance of PSI in oe*STN8*, the photochemical quantum yield of PSI (Φ_I_) and the quantum yield of non-photochemical energy dissipation in PSI due to donor (Φ_ND_) or acceptor side limitation (Φ_NA_) were determined in WT, oe*STN8, stn8-1* and *stn7 stn8* plants by performing a light curve with increasing light intensities (Figure S2). All PSI values determined for all mutant lines, except *stn7 stn8*, lay within the standard deviation of the WT. Only at weaker light intensities up to 216 μmol photons m^−2^s^−1^ did oe*STN8* show a slight tendency to higher (and *stn8-1* to lower) Φ_I_ values compared to WT (Figure S2).

### Increased STN8 activity results in higher grana stacks

**Figure 8:**
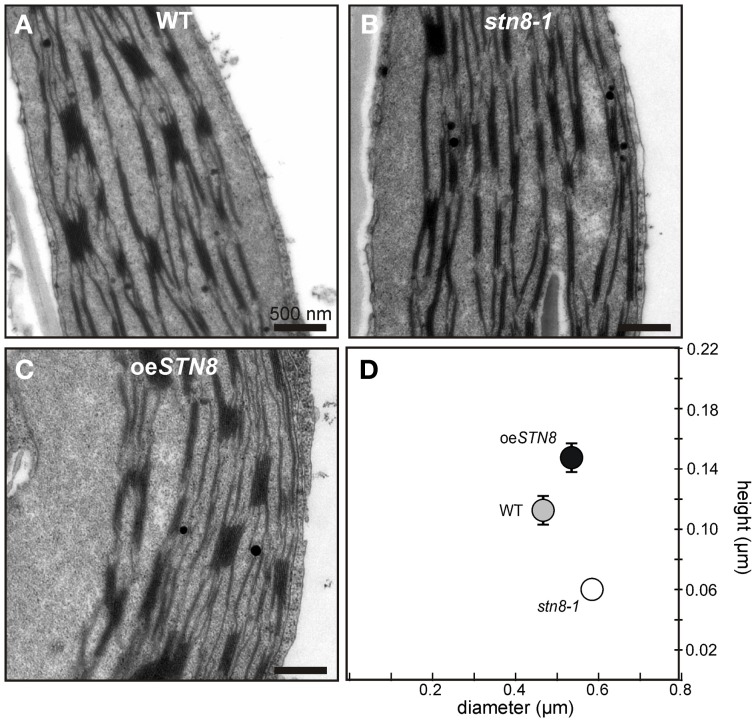
**Altered STN8 levels affect thylakoid ultrastructure. (A–C)** True leaves of 4-week-old WT (Col-0), *stn8-1* and oe*STN8* plants grown at 100 μmol photons m^−2^s^−1^ at a 12/12 h night/day cycle were fixed 2 h after the onset of light and analyzed by transmission electron microscopy (TEM). Representative cross-sections of chloroplasts are shown for WT **(A)**, *stn8-1* **(B)** and oe*STN8* **(C)**, respectively. Bars represent 500 nm. **(D)** Scatter plot of the average grana diameter and height values (*n* = 30) in the genotypes shown in **(A–C)**. Bars represent the respective standard errors of the mean (SEM).

PSII phosphorylation, primarily mediated by STN8, has been proposed to alter the macroscopic folding of the thylakoid membrane (Fristedt et al., 2009). Specifically, a reduction in thylakoid protein phosphorylation, as in *stn8-1*, is associated with an increase in grana diameter (Fristedt et al., 2009). This effect was even more pronounced in the *stn7 stn8* double mutant (Fristedt et al., 2009; Herbstova et al., 2012; Armbruster et al., 2013), which in addition has fewer membrane layers per granum and therefore shows a reduction in grana height (Armbruster et al., 2013). To determine the effects of enhanced STN8-mediated phosphorylation on thylakoid ultrastructure, WT, oe*STN8* and *stn8-1* plants were adapted to LL for 1.5 h after 12 h of darkness, and the chloroplasts in thin sections of leaves were analyzed by transmission electron microscopy (Figures 8A–C). While grana stacks of WT exhibited an average ratio of diameter to height of 0.47/0.11 (±0.01/0.01) μm, the corresponding value for *stn8-1* grana was 0.58/0.06 (±0.02/0.00) μm (Figure 8D). These observations confirmed the significant tendency toward an increase in grana diameter in *stn8-1* (Fristedt et al., 2009), and the detected decrease in grana height is in good agreement with the measurements by Armbruster et al. (2013), giving a diameter/height ratio for *stn8-1* that lies between those for WT and *stn7 stn8* double mutant plants. oe*STN8* on the other hand showed an average grana diameter/height ratio of 0.54/0.15 (±0.01/0.01) μm (Figure 8D). The moderate but significant increase in grana height compared to WT is in accordance with that seen in other mutant lines (i.e., *tap38*) showing enhanced thylakoid phosphorylation (Armbruster et al., 2013). However, this increase in grana height does not seem to be compensated by a decrease in grana diameter. All differences in grana diameter and height between the lines are significant at *p*-values of <0.05. Whether the observed changes in thylakoid ultrastructure associated with increased levels of STN8 result from (i) enhanced PSII phosphorylation, (ii) the concomitant alterations in PSII supercomplex composition or (iii) possible changes in the phosphorylation state of the CURT1 proteins, which were recently shown to mediate grana formation by inducing membrane curvature (Armbruster et al., 2013), remains to be elucidated.

## Discussion

### STN kinases: similar biochemical features but no functional interdependence

When STL1, the STN8 homolog in *Chlamydomonas reinhardtii*, was shown to be phosphorylated in an Stt7-dependent manner (Lemeille et al., 2010), the idea was proposed that STN7 and STN8 might also form part of a kinase cascade (Lemeille and Rochaix, 2010). It was later shown that the phosphorylation status of STN7 is not affected in the *stn8-1* mutant, indicating that STN7 is not phosphorylated by STN8 (Reiland et al., 2011). Therefore, it remains unclear whether STN7 and STN8 interact functionally, either directly or indirectly. It is however accepted that there is some substrate overlap between the two (Bonardi et al., 2005; Tikkanen et al., 2008, 2010), and this also applies to the corresponding protein phosphatases, with TAP38/PPH1 potentially targeting STN8 substrates (Vainonen et al., 2008; Pribil et al., 2010) and overexpression of PBCP - the PSII core protein phosphatase - affecting state transitions (Samol et al., 2012). Here, we show that, despite this complex interplay of these two protein kinase/phosphatase couples, knockout or overexpression of one STN kinase does not significantly affect the activity or steady-state level of the other (Figure 1). Therefore, the overlap in substrate specificity of STN7 and STN8 is not reflected in a reciprocal response to changes in the level of either protein.

While the topology of Stt7 has been clarified experimentally (Lemeille et al., 2009), the assumption that STN8 is a single-pass transmembrane thylakoid protein with its kinase domain facing the stroma has been based solely on sequence predictions and the inference that PSII proteins are phosphorylated exclusively on their stromal moieties (Vainonen et al., 2005). However, the predictions of the putative transmembrane domain (TM) are not clear-cut, with some algorithms for TM prediction (i.e., TMHMM and SOSUI) suggesting that STN8 is a soluble protein. We have now confirmed that STN8 is an intrinsic thylakoid membrane protein, very similar in character to STN7 (Figure 2).

Furthermore, this study has also addressed the question whether STN7 and STN8 exist as monomeric enzymes or are associated with HMW complexes, as previously shown for Stt7 under state 1 and state 2 conditions (Lemeille et al., 2009). In 2D BN-/SDS-PAGE analyses we show that both STN kinases were associated with HMW complexes, and their presence in multiple assembly states of high molecular weight was further supported by data from sucrose-density-gradient centrifugations (Figures 3, S1). Interestingly, alterations in light quality had no significant effects on the localization of the STN kinases within the thylakoid membrane (Figure 3A). Thus, there is now comprehensive evidence that both STN7 and STN8 operate in close association with the major photosynthetic complexes. A lack of STN8 accumulation in plants devoid of PSII complexes (Figure S1) might further reflect the necessity for a close spatial relationship between STN8 and PSII core proteins, its most likely substrates. In line with these findings, STN8 was mainly detected in grana stacks or grana margins, the thylakoid fractions in which PSII accumulates (Figure 3A). These observations suggest that phosphorylation of PSII subunits by STN8 occurs directly. However, a kinase cascade residing in close proximity to the PSII complex cannot be ruled out. The latter scenario would explain results obtained by Hou et al. (2003), who showed that washing thylakoids with 2 M NaBr led to loss of the capacity for PSII core protein phosphorylation, whereas we showed here that most of the STN8 protein remained bound to the membrane after similar treatments (Figure 2B). Therefore, it is tempting to speculate that, instead of affecting the presence or activity of STN8, washing with NaBr removes downstream components that are part of a putative PSII core phosphorylation cascade.

### The STN kinases exhibit distinctly different regulatory features

It is generally accepted that STN7 activity can be inhibited via the stromal ferredoxin-thioredoxin pathway, but the precise site of inactivation remains a matter of speculation. Two main scenarios have been considered: (i) thioredoxins directly target the stroma-exposed cysteines Cys 187 and Cys 191, which reside within the ATP binding pocket (Puthiyaveetil, 2011) and (ii) the redox signal is transferred to the lumen via the putative CcdA/Hcf164 pathway targeting the lumenal cysteines Cys 65 and Cys 70 (Lemeille and Rochaix, 2010). Here, we have presented experimental evidence for a direct physical interaction between STN7 and recombinant thioredoxin-f (recΔ TRX-f), which is not affected by replacement of the luminal STN7 cysteines Cys 65 and Cys 70 (Figures 4B,C). This observation supports the idea that STN7 is targeted by thioredoxins at its stromal CxxxC motif, which presumably interferes with the binding of ATP to the ATP binding pocket, especially under HL conditions, and thereby leads to the inactivation of STN7. The lack of this stromal cysteine motif in Stt7 and the fact that no thioredoxin-dependent inhibition of Stt7 has been found in *C. reinhardtii* (Puthiyaveetil, 2011) both argue in favor of this scenario. Further evidence for disulfide bridge formation in STN7, involving cysteines other than the known N-terminally located ones, was provided by redox titration experiments, which showed that STN7 reduction results in a shift in its migration behavior even in the absence of the N-terminal cysteine motif (Figure 4A). This suggests the presence of a second redox-sensitive motif besides the confirmed N-terminally located one, which could be regulated by thioredoxins. The observation that the electrophoretic mobility of STN7 is increased under reducing conditions is rather unusual for the opening of a disulfide bridge and might therefore be attributable to the release of an as yet unknown STN7-bound cofactor.

At least one of two conserved cysteine motifs, both of which are absent in STN8, is thought to be involved in thioredoxin-mediated down-regulation of Stt7/STN7 activity and protein levels under HL conditions (Rintamäki et al., 2000; Lemeille et al., 2009; Puthiyaveetil, 2011; Wunder et al., 2013). Thus, in contrast to STN7, STN8 activity is retained or even increased under these lighting conditions (Bonardi et al., 2005; Tikkanen et al., 2008).

As STN8 has none of the cysteine motifs that are commonly subject to redox-dependent regulation (Depège et al., 2003), it seemed possible that STN8 activity might be controlled on the level of protein amounts. However, in contrast to STN7, levels of the STN8 protein remained surprisingly stable both under activity-promoting and inactivating light conditions (Figure 5), suggesting that an activity-modulating mechanism must act on STN8. As such, reversible phosphorylation of STN8 could represent an essential mode of regulation. The phosphorylation of STL1, the STN8 homolog in *C. reinhardtii*, in a Stt7-dependent manner (Lemeille et al., 2010) argues in favor of this scenario.

### Physiological effects of varying STN8 protein levels

Elevated STN8 levels resulted in an overall increase in CP43, D1 and D2 phosphorylation under all light conditions tested (Figure 6A). Only FR conditions, which cause a strong oxidation of the PQ pool, led to a substantial decrease in PSII phosphorylation even in the presence of increased amounts of STN8, supporting the notion that redox-responsive mechanisms act (presumably indirectly; see above) on STN8 activity. This PSI light-dependent dephosphorylation of PSII core proteins was proposed to be relevant for the formation of PSII supercomplexes, the photosynthetically most efficient conformation of PSII (Tikkanen et al., 2008; Tikkanen and Aro, 2012). In contrast, under HL conditions, LHCII is preferentially released from the photosystems to participate in heat dissipation (Allakhverdiev and Murata, 2004; Murata et al., 2007; Allakhverdiev et al., 2008) and amounts of PSII supercomplexes are decreased due to increased PSII protein phosphorylation (Tikkanen et al., 2008, 2010). While studies on the role of PSII core phosphorylation in D1 turnover based on the protein kinase mutants *stn8* and *stn7 stn8* have led to somewhat contradictory results (Bonardi et al., 2005; Tikkanen et al., 2008; Fristedt et al., 2009), more recently the consensus has emerged that PSII phosphorylation exerts a rather indirect influence on PSII turnover via the modulation of thylakoid ultrastructure and PSII complex formation and migration (Grouneva et al., 2013). Here we show that the increased PSII core phosphorylation in oe*STN8* plants (Figure 6A) indeed leads to a slight reduction in photoinhibition after long-term exposure to fluctuating HL (Figure 7A). The underlying increase in D1 turnover efficiency can be ascribed to mechanisms that involve (i) PSII supercomplex disassembly and/or (ii) the modulation of thylakoid membrane stacking.

(i) While Tikkanen et al. (2008) interpreted the observed delay in D1 degradation in the *stn8-1* mutants under HL as a consequence of slower disassembly of PSII supercomplexes, no such changes in the ratio of PSII supercomplexes to PSII monomers could be detected by Fristedt et al. (2009) after 3 h of HL treatment. Interestingly, the direct comparison of PSII supercomplex formation in WT, oe*STN8* and *stn8-1* of this study revealed an obvious discrepancy between the respective genotypes under D and LL, and even more so under HL conditions (Figures 6B,C). While the disassembly of PSII supercomplexes was slightly promoted in oe*STN8*, PSII supercomplexes clearly accumulated in *stn8-1*, suggesting clear effects of altered STN8 levels. However, these observations do not fully explain the altered resistance to photoinhibition seen for oe*STN8* but not for *stn8-1* (Figure 7A), as both genotypes exhibit aberrant PSII supercomplex formation compared to WT.

(ii) The slightly higher resistance of oe*STN8* to photoinhibition could also be due to an increase in charge-dependent repulsion of the thylakoid membranes, which is reported to cause looser grana stacking and therefore allows faster lateral movement of proteins (Fristedt et al., 2009). In fact, compared to WT, slight changes in macroscopic thylakoid membrane folding could be observed in oe*STN8* under low light intensities (Figure 8), where differences in PSII phosphorylation between oe*STN8* and WT are only marginal (Figure 6A). Surprisingly, both height and diameter of the grana stacks seemed to be slightly increased in oe*STN8* (Figure 8), which argues against facilitated movement of membrane proteins between grana and stroma thylakoids, at least from the perspective of the grana diameter (Fristedt et al., 2009). The idea that the association of HL-induced PSII phosphorylation with altered grana stacking is purely coincidental, and that other HL-induced, STN8-independent processes are actually responsible for the modulation of grana stacking can probably be ruled out also, as oe*STN8* shows somewhat increased grana stacking already under LL (Figure 8). This provides evidence that thylakoid protein phosphorylation mediated by STN8 is indeed responsible for the observed changes in thylakoid remodeling. Nevertheless, it remains unclear whether STN8-dependent changes in grana formation actually result from alterations in PSII core protein phosphorylation or possible changes in the phosphorylation state of the CURT1 proteins, which were shown to mediate grana formation by inducing membrane curvature (Armbruster et al., 2013), or other so far unknown thylakoid components. In this respect, whether the differences in PSII supercomplex composition are the cause or the consequence of the altered macroscopic thylakoid membrane folding remains to be elucidated. In conclusion, it can be stated that the aberrant phosphorylation of PSII core proteins in oe*STN8* and *stn8-1* plants has only a minor impact on photosynthetic performance (Figures 7, S2).

## Author contributions

Research was designed by Tobias Wunder, Mathias Pribil and Dario Leister. Research was performed by Tobias Wunder, Wenteng Xu, Qiuping Liu, Gerhard Wanner, and Mathias Pribil. The manuscript was prepared by Mathias Pribil, Tobias Wunder, and Dario Leister. The whole study was supervised by Dario Leister.

### Conflict of interest statement

The authors declare that the research was conducted in the absence of any commercial or financial relationships that could be construed as a potential conflict of interest.
