# Supplementary material for: The major thylakoid protein kinases STN7 and STN8 revisited: effects of altered STN8 levels and regulatory specificities of the STN kinases
Source: Front Plant Sci. 2013 Oct 21;4:417. doi: 10.3389/fpls.2013.00417 (PMC3801152; doi:10.3389/fpls.2013.00417)
Supplement: Supplementary file 1 [file DataSheet1.PDF]

Supplementary Figures

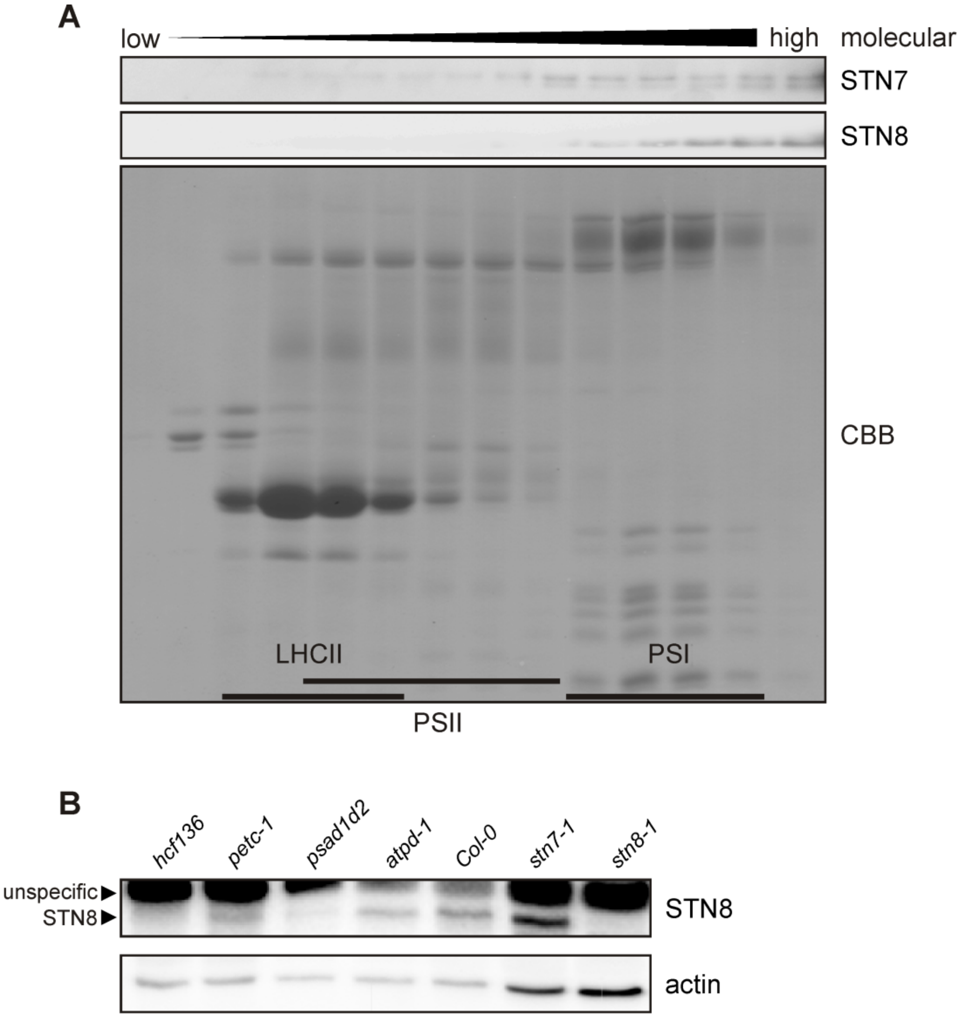

Figure S1 | Association of STN kinases with high-molecular-weight protein complexes and dependence of STN8 accumulation upon photosynthetic complexes.
(A) Analysis of STN7 and STN8 by sucrose-gradient centrifugation. WT thylakoids at a concentration of 2 mg chlorophyll/mL were solubilized with 1 % (w/v) β-DM and separated by centrifugation in a linear sucrose gradient. Gradient fractions (14) were loaded in order of increasing sucrose density, and specific antibodies for STN7 and STN8 were employed for immunolabelling. Loading was controlled by staining with Coomassie (CBB). **(B)** Total proteins were extracted from WT (Col-0), *stn7-1,* and *stn8-1*, and from mutant plants devoid of PSII (*hcf136*), functional Cyt *b6f* (*petc-1*), PSI (*psad1-1 psad2-1* = *psad1d2*) or ATP synthase (*atpd-1*). STN8 levels were analyzed with antibodies raised against STN8 after Western blotting, and actin served as a loading control. STN8-specific signals are marked by black arrowheads.

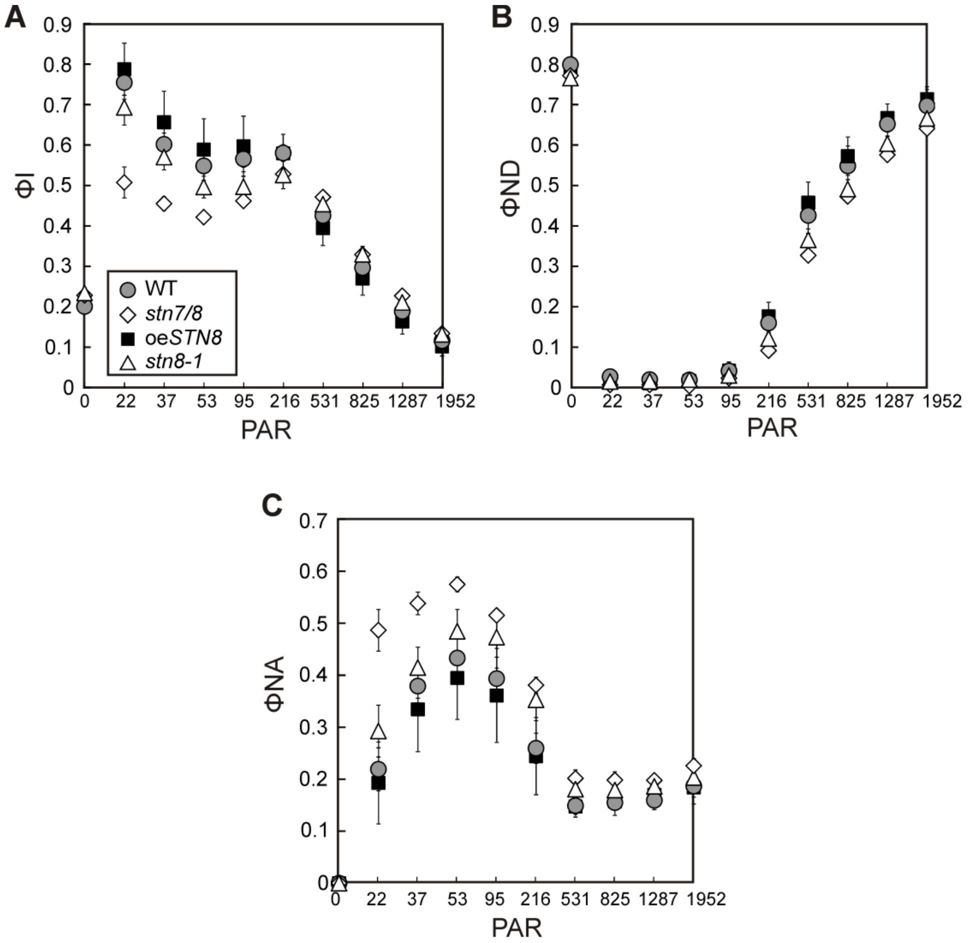

Figure S2 | Dependence of absorption parameters in WT (Col-0), oe*STN8*, *stn8-1* and *stn7 stn8* plants on light intensity

(A-C) Plants were grown under an 8 h/16 h day/night regime at 100 µmol photons $m^{-2}s^{-1}$. Average values (± SD) of five individual plants are shown. The photochemical quantum yield of PSI (ΦI) **(A)** and the quantum yield of non-photochemical energy dissipation in PSI due to donor-side limitation (ΦND) **(B)** or acceptor-side limitation (ΦNA) **(C)** were monitored as red light intensities were increased stepwise (22, 37, 53, 95, 216, 513, 825, 1287 and 1952 µmol photons $m^{-2}s^{-1}$, each lasting for 5 min) after 10 min of dark adaptation. PAR, photosynthetically active radiation (µmol photons $m^{-2}s^{-1}$); circles with grey filling, WT (Col-0); squares with black filling, oe*STN8*; triangles; *stn8-1*; diamonds, *stn7/8* double mutant.
